# Reconstructive surgery for severe breast asymmetry post-paediatric cardiovascular operation by anterolateral thoracotomy

**DOI:** 10.1093/icvts/ivad191

**Published:** 2023-11-24

**Authors:** Naohiro Ishii, Takanori Ko, Makoto Shimabukuro, Kyoichi Matsuzaki

**Affiliations:** Department of Plastic and Reconstructive Surgery, International University of Health and Welfare Hospital, Tochigi, Japan; Department of Plastic and Reconstructive Surgery, International University of Health and Welfare, School of Medicine, Chiba, Japan; Department of Plastic and Reconstructive Surgery, International University of Health and Welfare Hospital, Tochigi, Japan; Department of Plastic and Reconstructive Surgery, International University of Health and Welfare Hospital, Tochigi, Japan; Department of Plastic and Reconstructive Surgery, International University of Health and Welfare, School of Medicine, Chiba, Japan

**Keywords:** Anterolateral thoracotomy, Breast maldevelopment, Breast reconstruction, Paediatric cardiovascular surgery

## Abstract

Breast maldevelopment is a troublesome long-term complication of anterolateral thoracotomies, and several cases of this event have been reported. However, breast reconstruction in such cases has not yet been reported in detail, and solutions for managing this event remain unexplained. Herein, we present a case of breast maldevelopment after anterolateral thoracotomy, in which symmetrical breasts were successfully obtained by breast reconstruction based on autologous tissue transfer. The surgical approach prior to pleurotomy in anterolateral thoracotomy, particularly when performed on female infant patients, must be modified to prevent postoperative breast malformation.

## INTRODUCTION

Anterolateral thoracotomies are routinely performed in paediatric cardiovascular surgery, and subsequent breast maldevelopment is a long-term postoperative complication [1, 2]. During maldevelopment, the volume difference between the breasts may appear to be remarkable with age until breast development finishes, and patients may experience long-term mental pain. Breast reconstruction in such cases has not yet been reported in detail. Furthermore, solutions for managing this troublesome event remain unexplained.

This case report presents a case of breast maldevelopment after anterolateral thoracotomy in which symmetrical breasts were successfully obtained by breast reconstruction based on autologous tissue transfers and suggests a solution for this troublesome complication.

## CASE REPORT

**Figure 1:**
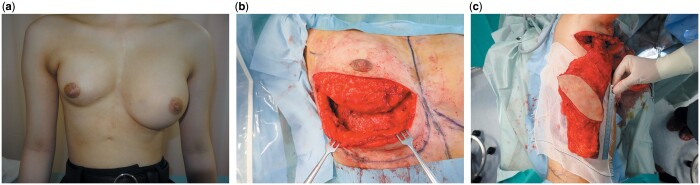
(**a**) Preoperative picture. Severe breast asymmetry is seen because of breast maldevelopment after paediatric cardiovascular operation. (**b**) Intraoperative picture after releasing breast contracture. (**c**) Intraoperative picture after harvesting extend latissimus dorsi musculocutaneous flap including skin paddle (6.5 cm × 18 cm) and adipo-musculocutaneous flap (10 cm).

A 16-year-old girl visited our outpatient department with severe breast asymmetry due to maldevelopment following paediatric cardiovascular surgery. She previously had Fallot tetralogy and underwent 2 thoracotomies, including a Blalock–Taussig shunt at 20 days of age and an intracardiac repair at 1 year of age. The intracardiac repair included ventricular septal defect closure by patching and monocuspid valve patch reconstruction of right ventricular outflow tract. Thereafter, maldevelopment of her right breast occurred as she grew older. A significant difference in volume (nearly 300 cm^3^) was found between the breasts; the right breast was pushed caudally, and the right nipple-areolar complex was located 2 cm more caudally than the left breast (Fig. 1a). The operative scar was located 1 cm above the inframammary folds and extended into the axillae. Magnetic resonance imaging showed no difference in volume between the pectoralis major muscles.

At 17 years of age, the patient underwent breast reconstruction using an extended latissimus dorsi musculocutaneous flap. A pectoral and axillary skin incision was made along the operative scar, extending to the anterior border of the latissimus dorsi muscle. We undermined above the pectoral major muscle and released breast contracture, following which the inferior border of the mammary gland moved to 4 cm below the skin incision (Fig. 1b) We harvested the flap as planned (Fig. 1c). The skin paddle was totally de-epithelized, and the flap was folded in 3 to make a high protrusion. The flap was transferred to cover the volume loss of the right breast without tension on the flap-feeding vessels.

**Figure 2:**
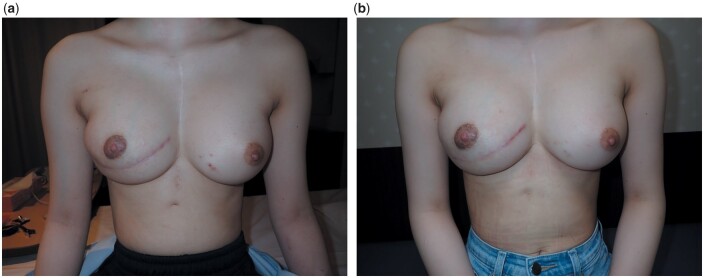
(**a**) Postoperative picture in 7 months after flap surgery. A slight asymmetry in lower volume of breasts was seen. (**b**) Postoperative picture in 3 months after fat grafting. Good symmetry in breasts was seen.

The postoperative course was uneventful, with no flap necrosis or infection. Breasts symmetry improved during the postoperative period; however, the lower volume of the right breast remained less than that of the left breast (Fig. 2a). We then performed fat grafting from the abdomen 7 months postoperatively. Harvested fat was centrifuged and grafted onto the lower part of the right breast via injection. The postoperative course was uneventful without infection. Good symmetry in the breasts was obtained through an extended latissimus dorsi musculocutaneous flap transfer and fat grafting (Fig. 2b).

## DISCUSSION

The skin incision of the anterolateral thoracotomy in paediatric patients is usually made at the intercostal space ranging from the 4^th^ to the 6^th^ and from the sternal edge to the scapular edge. This incision can cause maldevelopment of the mammary glands, mainly for the following 2 reasons [2]. First, the lower border of the paediatric mammary gland is 1.5 cm inferior to the inferior border of the areola; therefore, the surgical approach using this incision can separate the mammary gland and force the vascularity of the lower mammary gland to be insufficient. Second, the lower border of the female breast moves from the 4th intercostal space to the 7th or 8th intercostal space as it grows. Therefore, scarring caused by this incision can disturb this movement and breast development.

In the present case, a skin incision was made at the 4th intercostal space, ranging from the sternal edge to the axilla, and the affected breast was hypoplastic and dammed up at the operative scar. We suppose that many feeding vessels of the mammary gland may have been injured during paediatric surgeries, with the operative scar disturbing breast development. Consequently, for preventing breast maldevelopment, a skin incision in a thoracotomy should be made at the 7th or 8th intercostal space, and dissection prior to pleurotomy should be performed in such a way as to preserve as many feeding vessels of the mammary gland as possible.

While an anterolateral thoracotomy with a short incision may qualify as a minimally invasive approach, the same cannot be said for an anterolateral thoracotomy with an extensive incision like the one performed during the patient's cardiovascular surgery. Other surgical techniques, such as posterolateral thoracotomy or vertical right mid-axillary thoracotomy, prove effective in preventing postoperative breast maldevelopment as they do not entirely eliminate scarring on the anterior chest wall, thereby preserving future breast tissue [3, 4].

We successfully obtained good aesthetic results with breast reconstruction based on autologous tissue transfer. To prevent postoperative breast maldevelopment, reconsidering the surgical approach prior to pleurotomy in anterolateral thoracotomy, is advisable. Additionally, patients experiencing postoperative breast asymmetry may benefit from consultations with breast reconstruction surgeons.

## ETHICS STATEMENT

The manuscript is in compliance with the ethical requirements of authors’ institution (International University of Health and Welfare Hospital). Written informed consent was obtained from the patient for publication of this report and the accompanying images.

**Conflict of interest:** none declared.

## Data Availability

All relevant data are within the manuscript.
